# Editorial: Neurological, psychological and endocrine markers of eating disorders and obesity

**DOI:** 10.3389/fnut.2023.1289370

**Published:** 2023-10-13

**Authors:** Fernando Fernández-Aranda, Roser Granero, Susana Jiménez-Murcia

**Affiliations:** ^1^CIBER Physiology of Obesity and Nutrition (CIBEROBN), Carlos III Health Institute, Madrid, Spain; ^2^Psychoneurobiology of Eating and Addictive Behaviors Group, Neurosciences Programme, Bellvitge Biomedical Research Institute (IDIBELL), Barcelona, Spain; ^3^Clinical Psychology Unit, University Hospital of Bellvitge, Barcelona, Spain; ^4^Department of Clinical Sciences, School of Medicine and Health Sciences, University of Barcelona, L'Hospitalet de Llobregat, Barcelona, Spain; ^5^Department of Psychobiology and Methodology, Autonomous University of Barcelona, Barcelona, Spain

**Keywords:** neurological, psychological, endocrine, eating disorders, obesity

## 1. Eating disorders and obesity: converging issues

Eating disorders (ED) are severe mental disorders characterized by severe and persistent dysfunctional thoughts about food accompanied by bizarre impairing eating behaviors. The ED category comprises diagnostic criteria for split diagnostic subtypes, being the most common in clinical and general populations anorexia nervosa (AN), bulimia nervosa (BN), binge eating disorder (BED) and other specified feeding and eating disorders (OSFED) (1). Some of these conditions can occur in individuals who are underweight (AN), normalweight (BN) and overweight (BED). However, most of the patients report cognitive distortions in their body image associated to compensative conducts to promote thinness (compulsive exercise, restrictive eating, purging, vomiting, and laxative/diuretics misuse). With the progression of the disorder, the altered consumption or absorption of food lead to multiple harming correlates on the physical and psychosocial areas (2), as well as high likelihood of comorbidity with other psychiatric disorders (such as anxiety, depression and substance use disorders), disability and mortality rates (3, 4). The prevalence estimates within developed societies for ED is around 5% of the general population (5), depending on the ED subtype, and large increases in incidences have been reported worldwide during the lasts decades, and specially after COVID lockdown. This panorama points to the need of new etiological research to identify underlying mechanisms among clinical and population-based samples.

Obesity (OB) is a severe disease, defined as the excessive-needless fat accumulation with the consequence of risk to health, with a body mass index (commonly used to classify weight state and calculated the weight in kilograms divided by the square of the height in meters, kg/m^2^) equal or over 30 among adulthood (6). Prevalence of OB has grown to epidemic magnitudes in developed countries around all ages (from early childhood through to old age), as a result of energy imbalance based on diets with increased consumption of energy dense foods without equivalent increase of physical activity. Under this budget research line, studies have observed the association between some type of foods (such as ultra-processed, which provide large amounts of saturated fats and free sugars) plus the eating patterns/habits with the incidence rates of obesity (7, 8).

While OB was been traditionally considered separate to the ED spectrum, recent empirical research and systematic reviews suggest that this polarization is flawed (9, 10). The complex relationships between OB and ED can be represented by dynamic shared pathways, impacting on weight and eating related problems. The common multiple interacting factors can be visualized in dynamic networks-structures, which contribute to the onset and to the developmental trajectories in each individual: (a) biological components, including genetics, brain functioning, endocrine and metabolic systems; (b) psychological variables, such as personality profile, self-perception and body image; (c) social context, mainly cultural and social ideals centered on beauty, as well as the impact of the media; and (d) other individual variables, comprising physical and mental health state. Other common features associated to the progression of these disorders are comorbid adverse physical and mental health conditions, substantial impairment in global quality of life, stigma and high resistance to changet (11).

## 2. Biological markers of ED and OB: from genetic to neuropsychological markers

Evidence from etiological research suggests the existence of multiple biological markers related to the onset and progression of both ED and OB, including genetic and neuropsychological processes, but also shared brain neurocircuitry and metaboloic signals. The overall observation of studies including neuropsychological measures is the identification of dysfunction in the impulse-inhibitory control responses and the executive functioning (including deficits in attention, decision-making and set-shifting), and these impairments should contribute to the ED-OB severity at baseline (12–16), and also on the short- and long-term therapy outcomes (17–21). More specifically, results of genetic neuroimaging, functional and molecular studies observed dopaminergic alterations and decreased basal metabolism in the prefrontal cortex and striatum among OB patients, and increased activation of reward brain regions in response to palatable food cues among ED patients (22). Consistent regional group differences have been identified according with the diagnostic subtype (23), suggesting that restrictive eating styles (such as AN) lead to decrease in brain volume (24) and brain abnormalities involving dorsolateral prefrontal cortex, visual cortex, mesolimbic areas (striatum, amygdala, hippocampus, and cerebellum) (25). On the other extreme of the continuum (excessive eating styles, such as those characteristics in the bulimic spectrum conditions [BN, BED and OB]), neuroimaging research suggests that the reward neural system achieves a relevant impact, and that the excessive episodes of food consumption should be the consequence of the need of large quantities of food until perceiving satisfaction (26–28).

The empirical evidence obtained in the etiological studies based on functional and molecular neuroimaging has transboundary clinical implications for dealing with ED and OB. First, for developing reliable prevention plans and early detection of eating problematic behaviors (29, 30). Second, to phenotype high-risk of ED and OB by identifying the neurobehavioral basis of the food choices, eating styles and motivation processes (31, 32). And third, to improve treatment effectiveness with new therapy interventions, which combine neurobiological techniques with other psychological therapies (such as cognitive-behavioral treatments) (33, 34). These procedures have been developed with the aim to modify neural plasticity of food-related brain functions implied in the onset and severity of the unappropiated learned behaviors, and therefore to restore and/or optimize healthy cognitions and eating habits. Currently, diverse neurophysiological interventions have been tested as encouraging experimental treatment tools, including functional magnetic resonance imaging (fMRI), pharmacogenetic fMRI, real-time fMRI neurofeedback, positron emission tomography (PET), single photon emission computed tomography (SPECT), repetitive transcranial magnetic stimulation (rTMS), transcranial direct-current stimulation (tDCS), and functional near-infrared spectroscopy (fNIRS).

The aim of this Research Topic is to provide new empirical evidence regarding the underlying triggers of ED and OB. The study of Liu et al. identified targets of fucoidan for treating perfluorooctanoic acid associated obesity through the regulation of endoplasmic reticulum stress, using different systematic analytical procedures (such as network pharmacologic and bioinformatics). Zhang et al. assessed the swallowing function of patients with acute ischemic stroke and next developed a prognostic model for the need for nasogastric tube. The cross-sectional analysis conducted by Ribeiro et al. examined the association between the altered sweet taste perception (i.e., intensity and pleasantness ratings of sour, salt, sweet and bitter tastants, and taste thresholds assessed by electrogustometry) with obesity. Finally, Janssen et al. carried out a controlled-randomized trial employed magnètic resonance imaging to assess the impact of a mindful eating intervention (with a duration of 8-weeks) on striatal reward anticipation response. Overall, the results obtained in these studies coud be particularly useful for developing reliable assessment tools and evidence-based intervention plans (focused on the individual needs of patients, from a multidisciplinary perspective) and necessary to reduce the burden of disease.

## Author contributions

FF-A: Writing—original draft, Writing—review and editing. RG: Writing—original draft, Writing—review and editing. SJ-M: Writing—original draft, Writing—review and editing.
